# Polymer Sorbent Design for the Direct Air Capture of CO_2_

**DOI:** 10.1021/acsapm.3c03199

**Published:** 2024-03-30

**Authors:** Mark Robertson, Jin Qian, Zhe Qiang

**Affiliations:** a School of Polymer Science and Engineering, The University of Southern Mississippi, Hattiesburg, Mississippi 39406, United States

**Keywords:** CO_2_ removal, chemisorption, polymer sorbent, poly(ionic liquid), negative emission, amine groups

## Abstract

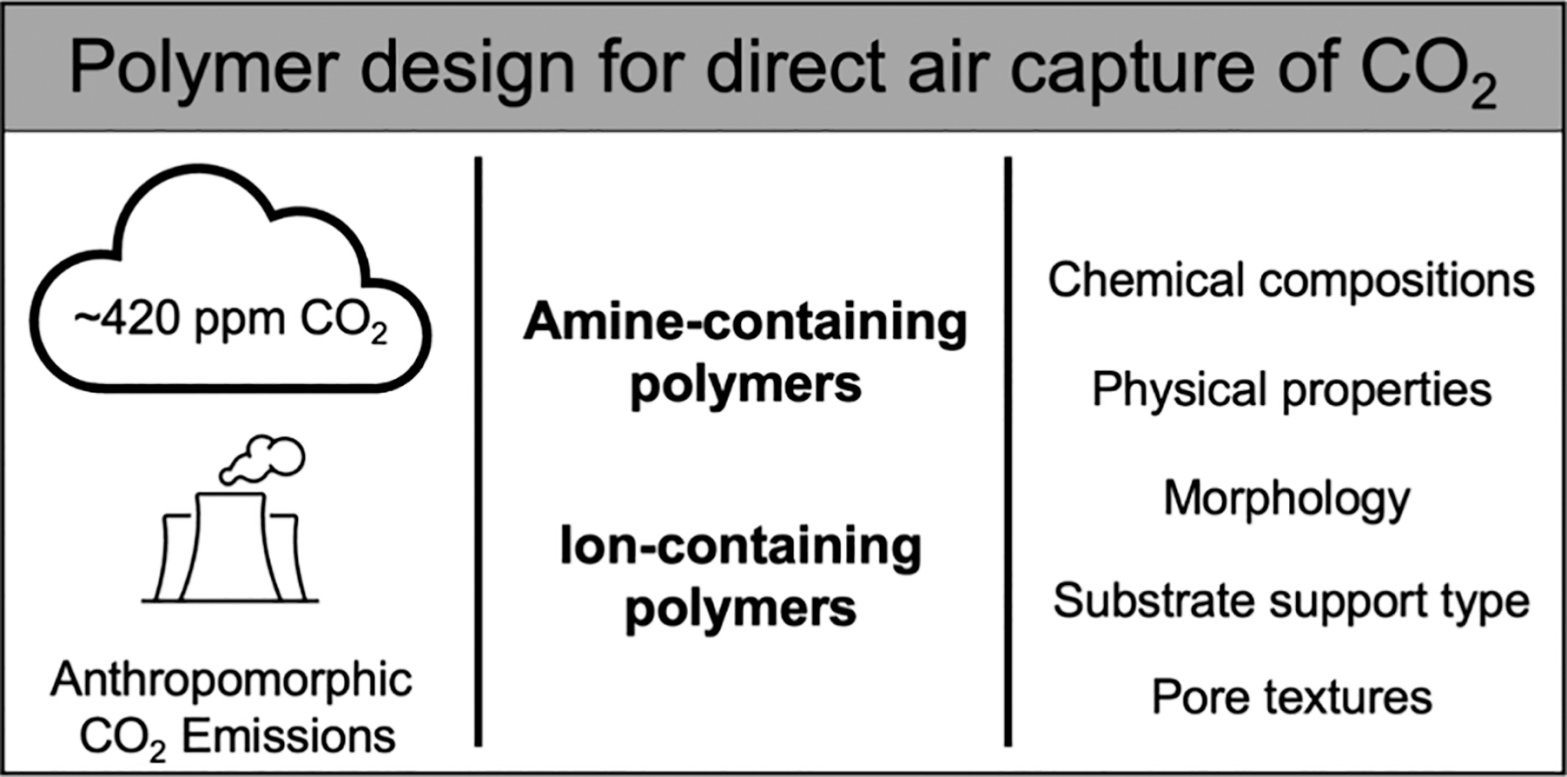

Anthropogenic activities have resulted in enormous increases in atmospheric CO_2_ concentrations particularly since the onset of the Industrial Revolution, which have potential links with increased global temperatures, rising sea levels, increased prevalence, and severity of natural disasters, among other consequences. To enable a carbon-neutral and sustainable society, various technologies have been developed for CO_2_ capture from industrial process streams as well as directly from air. Here, direct air capture (DAC) represents an essential need for reducing CO_2_ concentration in the atmosphere to mitigate the negative consequences of greenhouse effects, involving systems that can reversibly adsorb and release CO_2_, in which polymers have played an integral role. This work provides insights into the development of polymer sorbents for DAC of CO_2_, specifically from the perspective of material design principles. We discuss how physical properties and chemical identities of amine-containing polymers can impact their ability to uptake CO_2_, as well as be efficiently regenerated. Additionally, polymers which use ionic interactions to react with CO_2_ molecules, such as poly(ionic liquids), are also common DAC sorbent materials. Finally, a perspective is provided on the future research and technology opportunities of developing polymer-derived sorbents for DAC.

## 1 Introduction

Since the Industrial Revolution, the rate of CO_2_ being released into the atmosphere has increased significantly due to rising energy demands and intensive human activities;^1^ the atmospheric concentration of CO_2_ has now reached approximately 420 ppm (parts permillion),^2^ while in the mid-1700s, prior to the Industrial Revolution, it was only ∼280 ppm. The anthropogenic release of CO_2_ is primarily associated with the combustion of fossil fuels for energy production, chemical manufacturing, and agricultural practices.^3^ The increased atmospheric CO_2_ concentration leads to higher average global temperatures and more severe weather events, due to greenhouse effects that can have disastrous consequences for global ecosystems and society.^4−8^ In a recent technical report from the Intergovernmental Panel on Climate Change (IPCC),^9^ it has been projected that the atmospheric concentration could double by the year of 2100 if the proper measures have not been placed, which could result in further increased risks to our planet. Therefore, it becomes an urgent need to mitigate the adverse consequences of unnatural increases in CO_2_ concentrations, which can be achieved through two key pillars of activities toward a carbon neutral society: (1) Avoiding CO_2_ emissions through transitioning to sustainable energy sources like solar, wind, or alternative fuels, and the development of technologies that can prevent the escape of CO_2_ directly from the source of emission (power plants, production facilities, etc.), also known as point-source emissions. (2) Actively removing CO_2_ from the air to reduce the current atmospheric CO_2_ concentrations.^10^ Over the past decades, an enormous amount of effort has been dedicated to developing sustainable energy sources,^11−13^ as well as technologies for the removal of CO_2_ from both industrial processes and the atmosphere; the later one is known as direct air capture (DAC).^14−20^

Initial technologies for preventing the runaway increase of atmospheric CO_2_ concentrations were through the development of point-source sorbents to scrub excess CO_2_ from process streams before they enter the environment, which have been established for nearly a century at this point.^21^ These conventional technologies typically rely on liquid sorbents, most commonly aqueous amines, to react with CO_2_ through a chemisorption mechanism in order to prevent its escape into the atmosphere. After chemisorption, the aqueous amine system can be regenerated through the application of heat to release absorbed CO_2_, allowing their store and/or use in further applications. However, with the further intensifying rate at which CO_2_ is released into the environment, solely scrubbing point-source contamination is not sufficient to mitigate increases in atmospheric CO_2_ concentration; the development of DAC processes has become critical. Unfortunately, the high heat capacity and associated energy cost for regeneration of aqueous amines make their use impractical for DAC, and the design of new materials for these processes has been at the forefront of research for the past two decades. It has been demonstrated that solid porous sorbents, like porous carbons, zeolites, or metal organic frameworks (MOFs), can uptake CO_2_ through favored interactions, which are generally benefited from tunable surface chemistries and potential for large-scale production. For example, Yuan et al. recently demonstrated the synthesis of high surface area, nitrogen-doped porous carbon sorbents through polycondensation of nitrogen containing precursors and potassium salts.^22^ The nitrogen species within the carbon frameworks can strongly interact with CO_2_ molecules to enhance the adsorption capacities of the sorbents, reaching up to 4.16 mmol/g at 100 kPa in pure CO_2_ source. While this study demonstrates a sorbent exhibiting a high adsorption capacity, atmospheric concentrations of CO_2_ are multiple orders of magnitude lower than this concentration, making it very challenging to translate these sorbents for DAC use. Additionally, Zaworotko et al. performed a benchmark DAC study on many common sorbent materials, including multiple zeolites and MOFs, through conducting mass spectrometry experiments under atmospheric CO_2_ concentrations.^23,24^ Across 14 sorbents which relied on the physisorption mechanism, their adsorption capacities were all very low, which are largely due to the low affinity between sorbent surface and CO_2_ molecules, as well as the competition between the uptake of CO_2_ versus water. However, it is worth noting that the physical properties of these materials can be tuned by additional functionalization methods, such as through surface engineering or altering chemical composition to reduce adsorption of water from the atmosphere in order to improve the DAC performance efficiently. As an example, zinc-based Calgary Framework 20 (CALF-20) MOFs contain hydrophobic pore surfaces,^25−27^ which result in high CO_2_ sorption capacities and the ability to withstand humid conditions without performance degradation in flue gas conditions (operated up to > 450 000 cycles). However, their ability to adsorb CO_2_ directly from the atmosphere remains unclear. Conversely, sorbents that employ a chemisorption mechanism can readily perform air capture with good selectivity through reactions between functional groups within the sorbent and CO_2_ molecules, making them some of the most promising materials for DAC technologies; several of them have already been incorporated into industrial systems. To provide a few examples, Global Thermostat, Climeworks, Carbon Engineering, and many other companies have developed various technologies to remove CO_2_ from the atmosphere relying on chemisorption processes. In many of these technologies, sorption process occurs through reactions between CO_2_ and amine functionalities within the sorbent,^28,29^ while ionic functionalities,^30,31^ like quarternary ammonium ions, can also be employed to interact with CO_2_ molecules. Similar to aqueous amines that have been used for point-source CO_2_ capture, DAC sorbents can be regenerated through various methods, while the ability to maintain performance after multitudes of cycles is critical for their adoption in practical applications. To provide further context, Lackner et al. performed cost analysis studies on a variety of common DAC sorbents.^14^ For most common chemisorption-based sorbents exhibiting moderate performance, including amine-loaded porous materials, a maximum budget of ∼$30 USD/kg, in conjunction with the ability to cycle roughly 100 000 times is required to make them become economically viable technological solutions. While the production cost of these materials is likely above $30 USD/kg, higher adsorption capacities and/or the ability to operate for more cycles would be required for them to be incorporated into practical solutions. Over the years, the development of novel polymers and polymer-derived materials for addressing sustainability needs is a major field of research.^32−34^ Importantly, the design of polymers for the chemisorption of CO_2_ has played an integral role in developing DAC technologies as these materials can exhibit satisfactory performance stability toward regeneration, while maintaining high sorption capacities and CO_2_ selectivity. We note that Global Thermostat provided one of the first demonstrations of using polymer materials for DAC in 2007 through a patent application detailing amine-containing materials grafted to large surface area porous materials;^35^ a similar approach was also investigated by Jones et al., who developed methodologies for the synthesis of hyperbranched aminosilicas for DAC.^36^ Early works in this area have sparked numerous investigations into development of many polymeric materials for the direct removal of CO_2_ from the atmosphere, including amine-containing polymers that are incorporated into solid supports through physical impregnation, covalent grafting, or in situ polymerization, as well as polymers that rely on ionic interactions for CO_2_ adsorption.^37−39^

**Figure 1:**
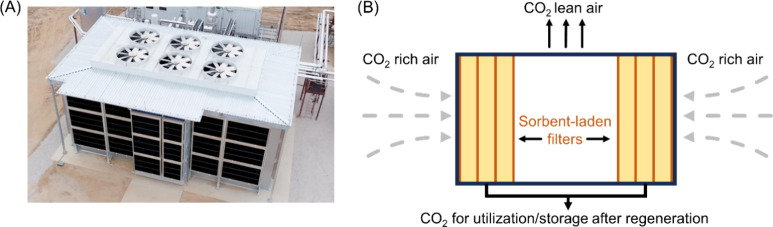
(A) Image of an air contactor developed by Global Thermostat for their DAC processes. (B) Generalized schematic of the operations within an air contactor which relies on solid sorbents for the capture of CO_2_ from the atmosphere.

While the rational design of active material for DAC is critical, it is also worth discussing the development of large-scale systems, which house the sorbents to carry out the adsorption/desorption processes.^40^Figure 1 depicts an image of a DAC air contactor developed by Global Thermostat using a solid sorbent system, as well as a generalized schematic of air contactors used for DAC. Generally, DAC is performed by directing the flow of atmospheric air into the contactor passively, or through directions from a series of energy efficient fans. In the case of DAC systems containing solid sorbents, such as those which rely on polymer materials, the air is transported across fixed beds that are loaded with sorbents, which can scrub CO_2_ from the air. Importantly, the design of the fixed sorbent bed can be instrumental in determining the efficiency of the DAC system as a whole. Careful design of these systems and sorbents involves considerations for achieving optimal external surface areas, mass transfer rates, and pressure drops to enhance the feasibility of broad adoption. For instance, the sorbent particle size and shape, as well as the sorbent bed void fraction, directly determine the pressure drop across the sorbent bed, which is also dependent upon flow rates of air throughout the DAC system among other factors. These aspects must be optimized in system design, which is an extensive field of research.^41−43^ After passing through the sorbent bed, the CO_2_ lean air is then released into the environment. Once the sorbent has become saturated, the adsorbed CO_2_ can be released through application of heat or steam and then directed toward other processes for utilization or storage. Generally, this occurs through a preliminary low temperature heating step that removes any adsorbed N_2_ to ensure high purity of the exiting CO_2_ stream. After the N_2_ is removed, a secondary higher temperature regeneration step is employed to desorb CO_2_ which is removed from the system through vacuum pumps. The high temperature and potential requirement for vacuum condition make the regeneration process potentially one of the most energy intensive steps for all DAC technologies. We would also like to emphasize that there are still multiple potential challenges associated with the widespread adoption of DAC technologies for CO_2_ removal. First, most large-scale operations for DAC require significant amounts of energy cost to operate the required machinery. If fossil fuels are the only available energy source, then this leads to reduced viability of the process for CO_2_ removal. However, multiple commercial processes that are in operation today can rely on renewable energy sources, such as geothermal and wind energy, and access to these burgeoning energy sources could be expected to increase in the near future. Additionally, successful adoption of DAC systems requires the development of infrastructure to either transport or store CO_2_ after removed from the atmosphere; these processes could also potentially be energy intensive. Many methods of CO_2_ storage have been demonstrated such as through injection into underground geological formations and/or mineralization, while there is also considerable research being performed on technologies for the integrated capture and conversion of CO_2_ directly into useful products.^44−47^ To remove significant amounts of CO_2_ from the atmosphere, the physical footprints of DAC capture systems can be exceptionally large. These factors along with techno-economic and life-cycle analyses of large-scale DAC have been discussed in multiple excellent reviews.^48−50^ Collectively, the fate of captured CO_2_, whether it is sequestered, repurposed, or utilized in various applications, coupled with the energy and material inputs for DAC process, play a pivotal role in determining the environmental impact of the entire operation. This knowledge is critical in establishing whether the DAC process contributes to a net reduction in atmospheric CO_2_ levels (i.e., negative emissions). A comprehensive understanding about economic and environmental benefits, in conjugation with strategic system design are also essential for ensuring that the DAC process not only captures CO_2_ efficiently but also aligns with sustainability goals, including minimizing carbon footprint and enabling the beneficial use of captured carbon sources.^51,52^

While there are many excellent reviews in the area which extensively discuss the application and performance of polymer-based sorbents for DAC,^53−57^ the intent of this article is to delve into the core aspects of polymer chemistry and physics, particularly emphasizing crucial molecular design parameters such as molecular weight, functional group identity, and diffusion characteristics. In turn, this article can encourage more polymer researchers to enter this important research area for addressing a critical environmental challenge. Specifically, we first provide a detailed overview of polymer design guidelines for the development of DAC technologies via sorption-based methods. We aim to not only summarize recent progress but also to stimulate interest in further exploration of both the underlying scientific principles and the development of practical technologies in this domain. Our discussion is separated into two major parts based on the materials sorption mechanism against CO_2_, including interactions with amine and ionic groups, respectively. Within these sections, design criteria to enhance different aspects of polymer-derived DAC technologies like adsorption capacity and cycle stability are discussed. Polymer chemical identity and physical characteristics, as well as type of support materials all influence the ability of DAC sorbents to interact with CO_2_, which are also discussed in this article. Furthermore, brief comments on the future outlook of DAC technologies and relevant research opportunities are provided. Considering the important role and emerging significance of DAC technologies toward achieving a decarbonized society, we anticipate a strong increase in research interest and advancements in the coming years. Together, we hope this work provides an important perspective to inform the design of DAC polymers, as well as their challenges and opportunities for the development of a future carbon neutral society.

## 2 DAC by Amine-Containing Polymers

Amine-containing chemicals have played an important role for the development of CO_2_ capture technologies since the mid-1900s, due to their ability to reversibly react with CO_2_ molecules, allowing their use for a number of cycles. The initial technologies in this area were based on aqueous solutions of amines, such as monoethanol amine (MEA), methyldiethanolamine (MDEA), and 2-amino-2-methyl propanol (AMP).^58^ These amines exhibit excellent affinity for the chemisorption of CO_2_, but their regeneration requires the application of heat to drive the equilibrium for desorbing CO_2_. The established aqueous amine technologies are typically very energy intensive to regenerate and also have potential to volatilize or leach the amine species into the environment, limiting their ability to perform DAC. To improve the energy efficiency of sorbent regeneration, in addition to enabling higher adsorption capacities through enhanced mass transport and improved site accessibility, many works have incorporated liquid amines into solid porous sorbents (such as silica), or physically blended with polymer substrates,^59^ which lead to lower energy input requirement to reach sorbent regeneration temperatures, compared to their aqueous amine counterparts.^60^ Specifically, aqueous solutions containing 30% MEA have been demonstrated as having a specific heat capacity of roughly 4.0 J/(g*K). Impregnating a solid sorbent with MEA reduces the specific heat capacity to ∼2.1 J/(g*K) which is even further reduced to ∼1.6 J/(g*K) for a solid sorbent impregnated with a polymeric amine.^60^ While impregnating solid sorbents with small molecule liquid amines greatly reduces energy requirements for regeneration, they can still easily leach from the scaffold and are susceptible to degradation at elevated temperatures. To address this challenge, efforts have been focused on the development of polymers with high numbers of amine, providing a marriage between the excellent CO_2_ sorption capacity with the stability and processability of polymeric systems. In this section, we discuss the use of amine-containing polymers and how their characteristic features can affect different aspects of their performance as DAC sorbents. Specifically, this discussion includes the effects of functional group identity, polymer physical properties such as chain topology and molecular weight, as well as various cycle stability considerations. It is worth mentioning that the majority of research regarding amine-containing DAC polymer sorbents is focused on systems supported by a solid porous scaffold, while notes regarding self-supporting, inherently porous polymers are also provided at the end of this section.

### 2.1 The Effects of Amine Identity on Adsorption Capacity and Regeneration

**Figure 2:**
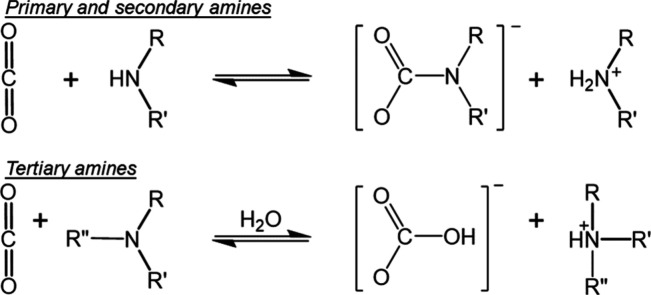
Reaction mechanisms of primary, secondary, and tertiary amines with CO_2_ during chemisorption.

Various polymeric amines sorbent have been synthesized with different backbones, amine group densities, and amine identities, including the most studied poly(ethyleneimine) (PEI) system,^61−64^ as well as other polymers such as poly(propyleneimine),^65−68^ and poly(allylamine).^69,70^ We note that PEI is by far the most investigated polymer for DAC applications, and will be a key focus of the following discussion. Generally, these DAC polymers can be physically impregnated, chemically grafted to, or polymerized within the pores of a solid porous sorbent, which provides structural stability and integrity, elevated surface areas to promote interactions with CO_2_ molecules, and lower the heat capacities to promote more energy efficient regeneration. It is also worth mentioning that other polymers with nitrogen containing functional groups, such as polyacrylamides and polyanilines,^71−73^ can perform DAC, yet primarily through physisorption-based mechanisms. The reaction of CO_2_ within amine containing polymers proceeds through the reaction mechanisms in Figure 2, which have been previously detailed in the literature.^74,75^

**Figure 3:**
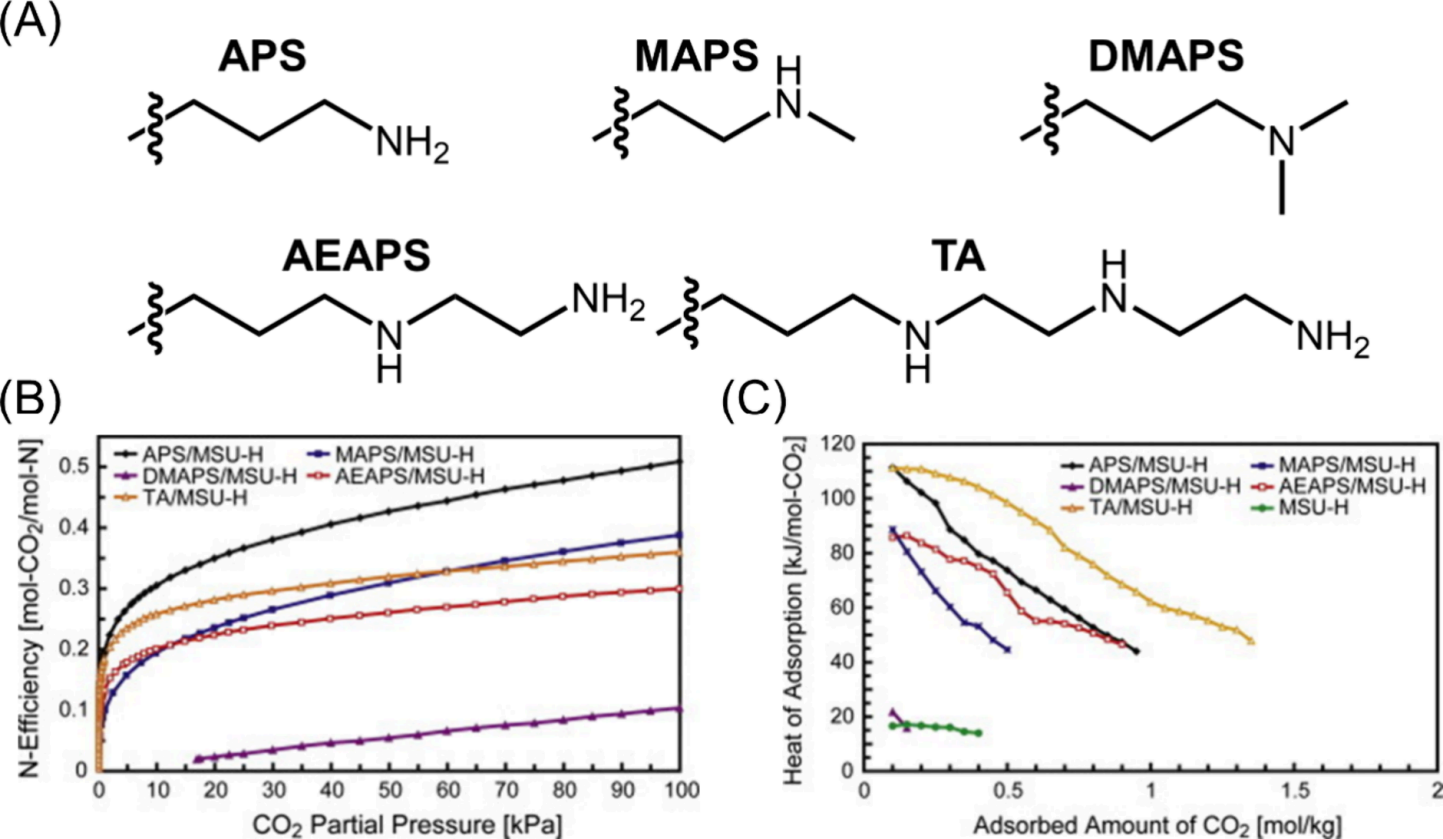
(A) Chemical structures of the amine studied by Yogo et al. (B) Amine efficiency and (C) calculated heats of adsorption for the corresponding amines. Reproduced with permission from Yogo et al.^82^ Copyright 2013 Elsevier.

For primary and secondary amines, chemisorption occurs through the formation of a zwitterionic intermediate requiring two amines to react in concert with a single CO_2_ molecule, ultimately forming an ammonium carbamate, which was detailed in two early works from Caplow and Danckerts.^74,75^ The formation of the ammonium carbamate product requires the presence of hydrogen groups to interact with the amine, so reactions between tertiary amines and CO_2_ typically involves water molecules through base-catalyzed formation of bicarbonates. This was described in an early work by Nguyen et al., who performed tracer ^14^C membrane transport studies on primary, secondary, and tertiary amines dissolved in aqueous solutions.^76^ Their results determined that the kinetics of the reaction between CO_2_ and the tertiary amines agreed well with a reaction between OH groups and CO_2_, indicating that the most probable reaction pathway was through hydrogen bonding between the amine and water molecules, which weakened the H–O bond of the water molecule, thus increasing its nucleophilicity. In turn, the water molecule could react with CO_2_, forming the products described in Figure 2. Multiple works have investigated the real-time progress of these reactions in many different amine-containing sorbents through in situ characterization methods, such as infrared spectroscopy, as well as solid-state nuclear magnetic resonance (NMR) spectroscopy.^76−81^ We note these reactions can occur under ambient conditions, making them excellent candidates for chemisorption-based DAC technologies. Generally, primary amines are more efficient for CO_2_ adsorption, likely due to steric effects from the additional alkyl substituent. Tertiary amines are the least efficient, requiring the presence of water and having weak affinities toward CO_2_. Yogo et al. studied the sorption performances of various small molecule amines suppored by silica substrates.^82^ Specficially, molecules containing primary, secondary, or tertiary amines were grafted to silica and their structures are provided in Figure 3(A). The amine efficiency of different sorbents are plotted in Figure 3(B), which shows that the molecule containing 1 primary amine prepared from aminopropyltriethoxysilane (APS) has the highest amine efficiency, followed by *N*-methyl-3-3aminopropyl-trimethoxysilane (MAPS), (3-trimethoxysilylpropyl)diethylenetriamine (TA), *N*-(2-aminoethyl)-3-aminopropyltriethoxysilane (AEAPS), and *N*-dimethyl-3-aminopropyltrimethoxy-silane (DMAPS), respectively. By comparing the results of the APS, MAPS, and DMAPs molecules, it can be observed that the amine efficiency decreases from primary to secondary and tertiary amines under identical CO_2_ partial pressure. Additionally, AEAPS and TA grafted silica had lower amine efficiency than APS due to the presence of the secondary amine lowering the efficiency of the sorbent. The reason corresponding to this altered efficiency is depicted by the heats of sorption provided in Figure 3(C). Briefly, the isosteric sorption heats were calculated from the sorption isotherms using the Clausius–Clapeyron equation, which has been established and used extensively for estimating heats of sorption. For the molecules containing a single amine (APS, MAPS, and DMAPs), the heat of sorption results shows that the primary amine (APS) has the highest value, indicating a more favorable interaction with CO_2_ followed by the secondary amine (MAPS) and tertiary amine (DMAPS). It is worth noting that average chemisorption heats of sorption range between 60 and 90 kJ/mol, further suggesting that the tertiary amine has very limited ability to react with CO_2_ molecules with the absence of water; the adsorption that occurred during these experiments is primarily due to physisorption based mechanism. Importantly, higher heats of adsorption are also associated with larger energy penalties for desorbing CO_2_ molecules to regenerate the sorbent, which must be considered in the design of the DAC material and will be discussed in a later section.

**Figure 4:**
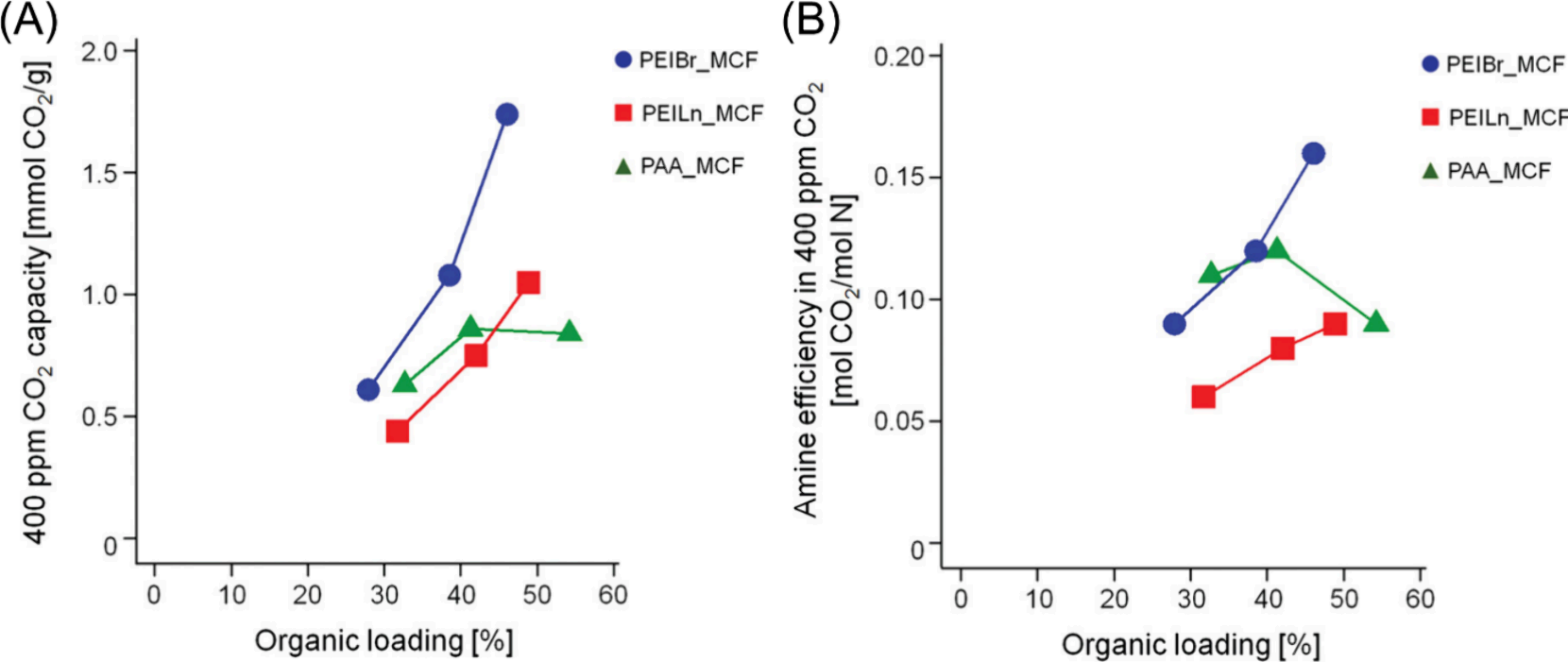
(A) CO_2_ adsorption capacities as a function of loading levels in branched PEI, linear PEI, and poly(allylamine). (B) Corresponding amine efficiencies for the sorbents determined from the amine content of each sorbent. Reproduced with permission from Jones et al.^83^ Copyright 2011 American Chemical Society

The impact of amine group identity on CO_2_ capture performance is also found in polymer materials. For instance, Jones et al. studied the ability of various aminopolymer loaded sorbents to uptake CO_2_ molecules from air.^83^ Specifically, branched and linear PEI materials containing both primary and secondary amines, as well as poly(allylamine), containing only primary amines, were impregnated within silica mesocellular foams and their sorption performance were determined based on aminopolymer loading levels. Figure 4(A) depicts the adsorption capacities of the amine-based polymer sorbents based on the amount of polymer incorporated into the mesocellular foams. The branched and linear PEI molecules have the highest adsorption capacities across all of the aminopolymer loading levels. However, after normalizing the adsorption capicities to the amine content within the polymers, it is found that the polyallyamine system has a higher amine efficiency than linear PEI and comparable to branched PEI at lower loading levels due to the sole presence of primary amines within the repeat units of the polymer.

**Figure 5:**
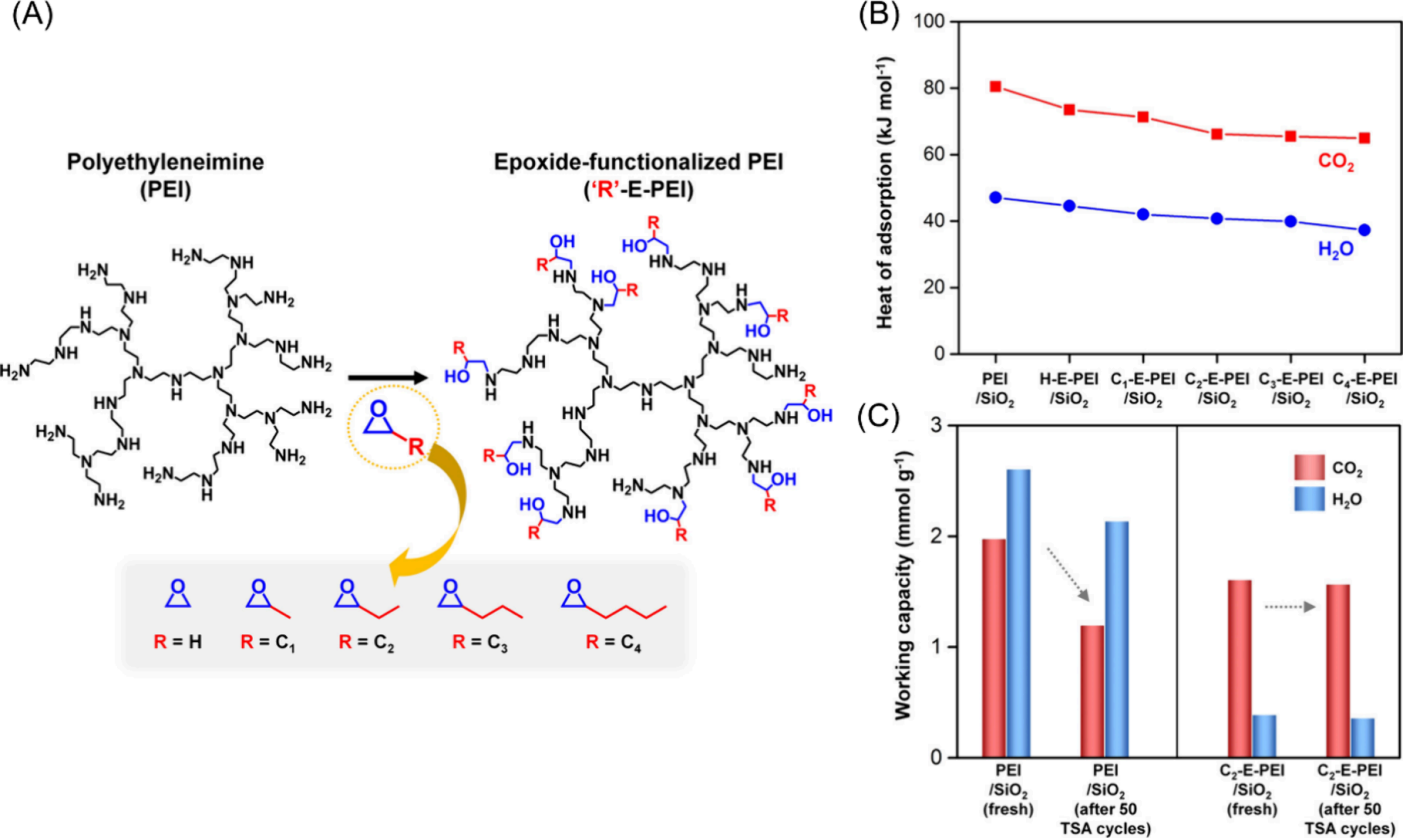
(A) Generalized scheme of functionalization branch PEI with various epoxide contaning moieties for the installation of hydroxyl groups at the primary amines of the polymer. (B) The effect of the functional group on the heat of adsorption of the sorbent for CO_2_ and water. (C) The effect of functionalization on the cycle stability of the sorbent after 50 cycles. Reproduced with permission from Choi et al.^84^ Copyright 2018 American Chemical Society.

In addition to the identity of the amine groups, their surrounding chemical environment in polymer backbones can play an important role in determining its ability to capture and release CO_2_ through altering the heat of adsorption. For example, Choi et al. investigated this phenomenon by functionalizing branched PEI molecules with various epoxide-containing molecules to produce hydroxyl moieties at varying distances from the amine functionalities within the polymer (Figure 5(A)).^84^ The functionalized, branched PEI polymers were impregnated within porous silica and their adsorption capacities were investigated as a function of the newly installed functional groups. Figure 5(B) depicts the impact of an epoxy-functionalization treatment of PEI on the heat of sorption of both CO_2_ and water. It is found that this postfunctionalization process leads to reduced heat of sorption, which further decreases with the installation of larger size functional groups resulting, and up to ∼34% energy savings for sorbent regeneration can be achieved. Importantly, a simultaneous reduction in the heat of sorption for water molecules, which directly competes with the uptake of CO_2_ from air, was also observed. These results were postulated to be due to two potential effects. First, the installation of electron withdrawing hydroxyl groups close to the amine lowers the basicity of the amine, thus reducing its affinity for CO_2_. Additionally, the decreased heat of adsorption with increased size of the functional group could be a result of higher steric bulkiness. The adsorption capacity of the neat PEI polymer and a functionalized polymer is shown in Figure 5(C). After 50 cycles, the performance of the neat polymer (PEI) diminishes signficantly, while the functionalized polymer remains almost unchanged. The improvement in perfrmance stability is due to the reduced number of amine groups, which mitigates the irreversible formation of urea (as one of the primary degradation pathways of amines for CO_2_ capture). By replacing the primary amine groups in polymers through post functionalization, the cycle stability is significantly increased, which is also aided by the additional steric bulkiness of the functional groups.^85^ Additionally, it has been reported that altering the chemical identity of the DAC polymer can provide an opportunity to increase cycle stability through preventing certain degradation mechanisms to occur. For instance, it was found that PEI can oxidatively degrade through chain rearrangement reactions to form six-membered rings that are easily oxidized into piperazinones.^86−88^ To address this issue, multiple works have found that changing the positions of the amines within the polymer backbone, using polymers like poly(allylamine) and poly(propyleneimine), can prevent the formation of degradation intermediates.^89−91^ While these polymers can suffer from reduced adsorption capacities due to reduced amine densities, they can exhibit much greater cycle stability. Collectively, these studies indicate the importance of the amine identity and surrounding environment on the performance of polymeric amine-derived sorbents. For the design of an optimal material for DAC, the affinity of the polymer against CO_2_ molecules should be balanced with its ability to be regenerated, which can be achieved through tuning the content of primary, secondary, and tertiary amines, while also including electron withdrawing neighboring functional groups to reduce the basicity of the amine.

### 2.2 The Effects of Polymer Characteristics on DAC Performance

**Figure 6:**
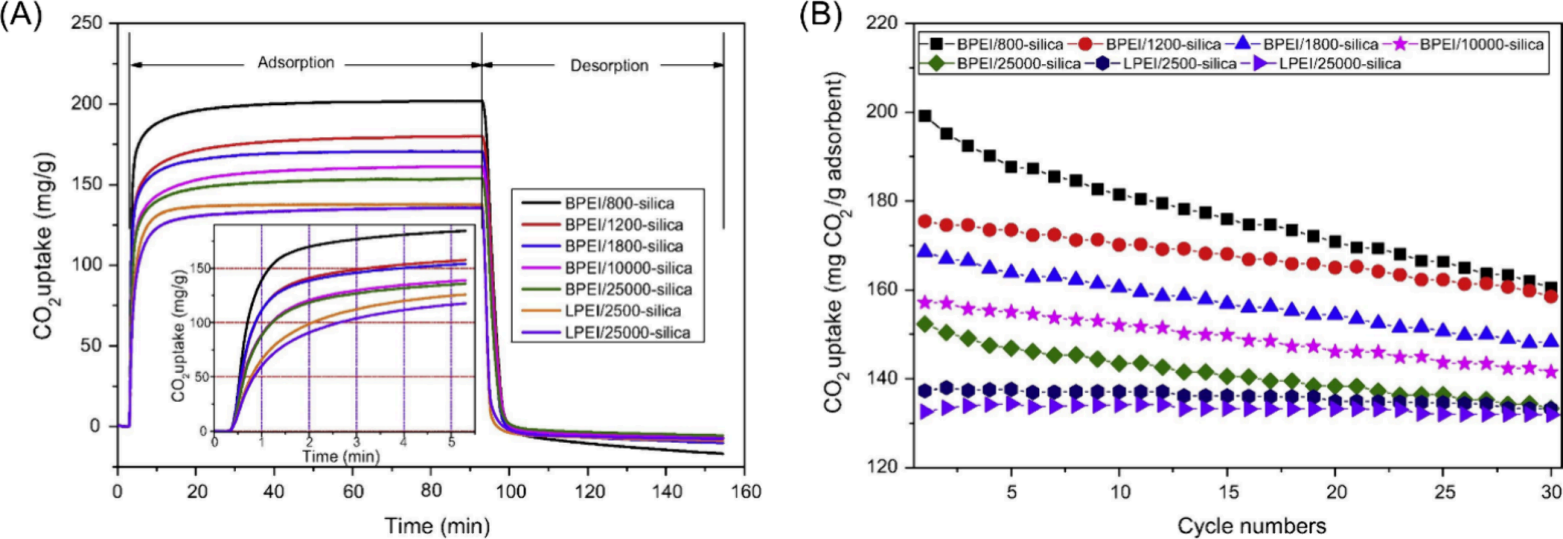
(A) Adsorption/desorption isotherms of PEI-loaded sorbents with varying molecular weight and chain topology. (B) Cycle stability of the PEI-loaded sorbents over 30 cycles. Reproduced with permission from Chen et al.^92^ Copyright 2014 Elsevier.

The molecular weight of polymers, their chain topology, loading levels, and the identity of the solid support directly impact polymer dynamics; these material parameters are obviously of critical importance when designing polymeric materials for the sorption of gas molecules such as CO_2_. Chen et al. investigated the effect of polymer branching and molecular weight on their CO_2_ adsorption performance, using a library of both branched and linear PEI polymers with different molecular weights ranging from 800 g/mol to 25 000 g/mol.^92^ The corresponding CO_2_ adsorption and desorption isotherms are presented in Figure 6(A); multiple trends in adsorption performance can be gathered from the sorption isotherms. In general, the branched polymers have enhanced adsorption capacities than the linear polymers, and the lower molecular weight polymers exhibit enhanced adsorption kinetics as well as higher adsorption capacities, compared to their higher molecular weight analogues; this work also indicated that the branched and lower molecular weight polymers are much more susceptible to performance degradation upon multiple sorption cycles. It was postulated that polymer branching can result in increased accessibility of primary amine groups, driving enhanced CO_2_ adsorption performance. Meanwhile, branched topology also affects the dynamics of the polymer which can also play a significant role in controlling its ability to adsorb CO_2_ through facilitating diffusion of the gas molecules within the polymer matrix. The enhanced dynamics of branched polymers has been demonstrated in multiple polymer systems. Analogously, Venkataramani et al. synthesized hyperbranched and linear polystyrenes of the same molecular weight and compared various characteristics to determine the effect of branching on the polymer properties.^93^ Importantly, the intrinsic viscosity of the branched polymer was greatly reduced in comparison to the linear polymer counterpart, as a result of more compact structure and reduced opportunity for chain entanglement, which collectively enhance polymer dynamics. This observation of enhanced dynamics in branched polymers is also found in poly(methyl methacrylate) systems.^94^ Increasing the molecular weight of polymers may lead to their decreased CO_2_ capacity due to the increased viscosity of the impregnated polymer matrix. However, increasing molecular weight of the polymers can improve the cycle stability of the sorbent. For all polymer sorbents shown in Figure 6(B), it is observed that increased molecular weight results in enhanced performance stability upon adsorption/desorption cycles and that the branched polymers exhibit much more pronounced performance degradation than linear polymers. We note that under the desorption conditions used in this study, the formation of urea is not observed, suggesting that the most possible degradation mechanism in this work is associated with leaching of the PEI from the sorbent.

**Figure 7:**
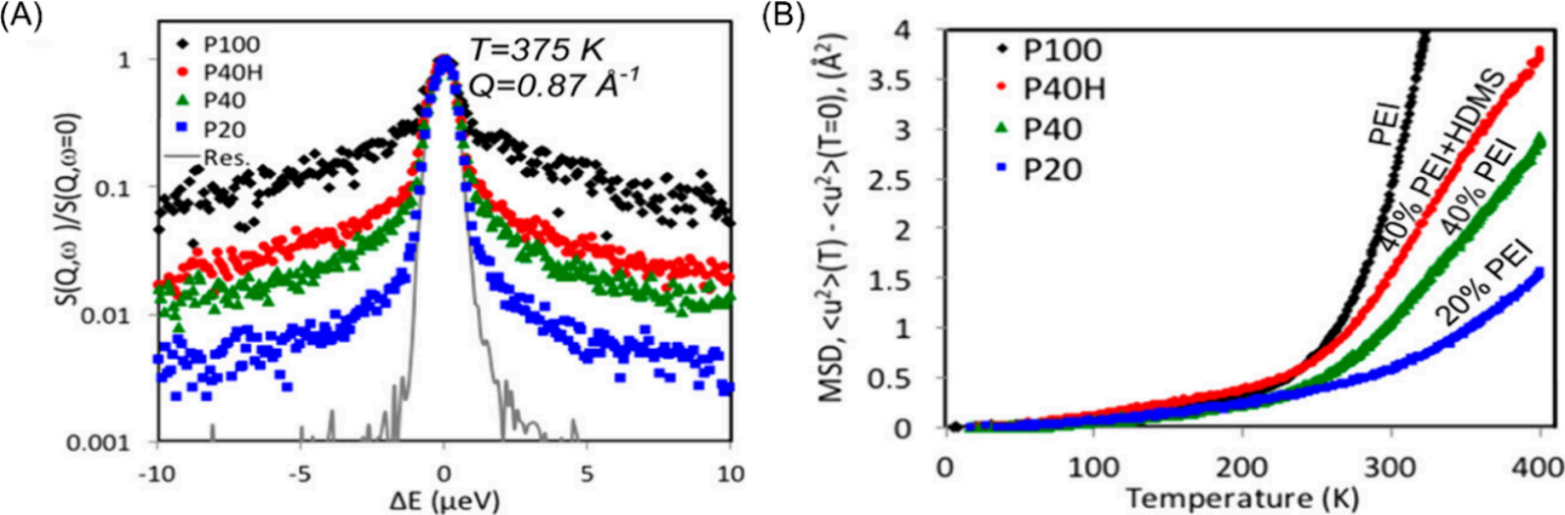
(A) Representative QENS spectra of PEI-impregnated mesoporous silica with varying loading levels and pore wall functionalization. (B) Mean-squared displacement for the sorbents calculated from QENS results. Reproduced with permission from Jones et al.^95^ Copyright 2017 American Chemical Society

These results suggest that polymer dynamics can play a key role in controlling the DAC sorption capacity of the materials, as well as their cycle stability. To further provide mechanistic understandings on this relationship, there are multiple seminal studies of understanding dynamics of PEI adsorbed within mesoporous silica. Specifically, quasielastic neutron scattering (QENS) was employed to study the polymer relaxations under confinement within the silica mesopores.^95−97^ Briefly, QENS is a robust, time-of-flight neutron scattering technique that enables the investigation of polymer dynamics in both space and time domains. For readers who are interested in learning more details about this technique, several papers employing QENS to study polymer dynamics, as well as a few key review articles are provided in the references.^98−102^ Some representative examples of QENS results for understanding how PEI loading levels, and the pore wall identity (or functionalization type) on impacting polymer dynamics are provided in Figure 7. Figure 7(A) the QENS spectra for PEI loaded into mesoporous silica at loading fractions of 20% (P20), and 40% (P40), in comparison to a bulk polymer (P100), and a sorbent where the silica support was functionalized with hydrophobic hexamethyldisilazane molecules (P40H). It is important to note that the loading fraction of the sorbent can directly impact the morphology of the adsorbed polymers, which also influences their sorption performance.^103^ To provide a brief background for understanding QENS spectra, the gray trace in Figure 7(A) is the resolution of the instrument. If all interactions between the incident neutrons and the polymeric samples were completely elastic, then no energy loss would occur, and the spectra would coincide with the instrument resolution. However, intra- and interchain polymer motions result in energy losses upon interactions between the incident neutrons and the sample, resulting in broadening of the spectra; the further these spectra deviate from the resolution of the instrument, the more enhanced dynamics are observed in the sample. These spectra can be used to extract useful information, such as the mean-squared displacement (MSD) of the samples, of which examples are provided in Figure 7(B). It is observed that with increasing temperature, the MSD experiences a distinct change in slope which can be associated with the onset of segmental motions within the polymer; the steeper the slope, the faster the dynamics within the sample. From the MSD results, it can be observed that the dynamics of the samples decrease from the bulk polymer, to P40 and P20. This is a result of strong interactions between the pore walls of the silica support and the hydrophilic PEI molecules greatly reducing polymer segmental mobility at low loading levels. When more polymer is loaded within the pores, the confinement contribution from the pore wall is less pronounced, and the polymer can exhibit enhanced mobility. On this end, when the silica pores are first functionalized with hydrophobic molecules, the dynamics can be greatly enhanced as evidenced when comparing the results of P40 and P40H. These results were then compared to the adsorption capacities of the sorbents, and it was found that enhanced adsorption capacities intuitively corresponded with enhanced polymer dynamics, which also agree with the adsorption performances found in many other works. With increased matrix dynamics, CO_2_ can more easily diffuse through the polymer matrix, thus promoting further adsorption within the polymer.

In addition to affecting the mobility of the polymer and, consequently, the adsorption capacity of the sorbent, the identity of the solid support used in these materials can impact their long-term cycling performance. Specifically, application of heat is the most common method for regenerating silica-supported DAC sorbents, as silica exhibits susceptibility to degradation when exposed to water or steam. However, at high temperatures (∼135 °C) urea can be formed from the amine precursors within the sorbent, effectively lowering the number of CO_2_ sorption sites for their subsequent use upon regeneration. This challenge can be addressed through altering solid support type, which has been demonstrated through the use of various support materials, such as mesoporous alumina, titania, and carbon. These porous supports can enable the regeneration of the sorbent at lower temperatures with the presence of steam, and help prolong the service life of the DAC sorbents. As an example, mesoporous alumina and SBA-15-derived sorbents (note SBA-15 is a common ordered mesoporous material, see refs (104−106)) have been systematically compared under accelerated steam stripping conditions of 105 °C for 24 h.^107−109^ The SBA-15 supporting DAC sorbents experienced 81% loss of its original adsorption capacity, whereas the mesoporous alumina-supported sorbent only decreased by approximately 25%. This result further suggests that the identity of the support can greatly enhance the application of DAC sorbents.

Moreover, formation of cross-linked networks in the polymer structure can assist in improving the performance of polymer derived sorbents for CO_2_ capture. As discussed in a previous section, polymers with lower molecular weights tend to have higher sorption capacities due to enhanced polymer mobility, which also inherently makes them less stable over a number of cycles. One method to increase the stability of the polymers is through incorporating chemical cross-links that prevent the polymer from leaching during sorption and/or regeneration. Jung et al. developed a method for synthesizing cross-linked PEI sorbents impregnated within mesostructured cellular foams from branched PEI and used 1,3-butadienediepoxide (BDDE) and epichlorodhydrin as cross-linkers.^110^ In comparison to neat PEI, cross-linked sorbents exhibited slightly reduced adsorption capacities. Noteworthily, in the case of the PEI sorbents cross-linked with BDDE, the sorbent exhibited only very limited performance decay after multiple cycles of regeneration at elevated temperatures due to their cross-linked nature, which promotes the thermal and chemical stability of polymer matrix. Furthermore, crosslinking polymer can also hinder the irreversible formation of urea functionalities which lowers the amine efficiency of the sorbent. A similar process has been employed by Wood et al. to synthesize macro-structured PEI sorbents that do not require a solid silica support. Specifically, they reported a method of cross-linking branched PEI with varied molecular weights using BDDE to form polymer gels.^111^ The gels were then physically ground into powders and employed as sorbents. This technique enabled the synthesis of cross-linked PEI powders with adsorption capacities that were dependent upon the amount of cross-linker introduced during synthesis. Additionally, optimal samples (molecular weight: 25 000 g/mol; BDDE concentration: 2 wt%) can exhibit excellent cycle stability over 10 cycles with virtually no performance loss after regeneration at 170 °C using microwave heating. A later work determined that a similar process using triglycidyl trimethylolpropane ether as an environmentally friendly cross-linker material can also enable excellent adsorption capacities and cycle stabilities with greater potential to be produced at a large scale.^112^ Furthermore, the production of cross-linked PEI sorbents with controlled macroscopic structures has also been reported by several groups. For example, Gray et al. synthesized cross-linked PEI fibers using *N*,*N*-diglycidyl-4-glycidyloxyaniline, which can capture up to 0.37 mmol/g of CO_2_ at DAC relevant concentrations.^113^ In this work, the cross-linker content could be increased to allow materials achieving ∼100% performance retention over cycles, although the sorption capacity of polymers for DAC was reduced due to the consumption of amines to form cross-links as well as reduced polymer mobility. For the sake of clarity, the polymers, their characteristics, and DAC performances of the references discussed within this section have been compiled into Table 1.

**Table 1:** Compiled List of Amine-Containing Polymers, Their Characteristics, and Performances

reference	polymer	amine type	molecular weight (g/mol)	polymer topology	solid sorbent identity	maximum adsorption capacity (mmol/g) @ 400 ppm CO_2_
83	PEI	2°	2500	linear	silica mesocellular foam	1.05
	PEI	1°, 2°, 3°	800	branched	silica mesocellular foam	1.74
	PAA	1°	1130	linear	silica mesocellular foam	0.86
(88)	PPI	2°	700	linear	mesoporous silica	1.25
	PPI	2°	1000	linear	mesoporous silica	1.3
	PPI	2°	6700	linear	mesoporous silica	1.25
	PPI	2°	36 000	linear	mesoporous silica	1.1
	PEI	2°	2500	linear	mesoporous silica	0.75
	PEI	2°	25 000	linear	mesoporous silica	0.75
(89)	PEI	1°, 3°	250	branched	mesoporous silica	2.5
	PPI	1°, 3°	350	branched	mesoporous silica	1.75
(96)	PEI	1°, 2°, 3°	800	branched	mesoporous silica	0.55–1.1
(103)	PEI	1°, 2°, 3°	800	branched	mesoporous silica	1.5
(107)	PEI	1°, 2°, 3°	800	branched	mesoporous silica	1.05
	PEI	1°, 2°, 3°	800	branched	mesoporous alumina	1.74
(109)	PEI	1°, 2°, 3°	800	branched	mesoporous alumina	1.71
(112)	PEI	1°, 2°, 3°	25 000	branched and cross-linked	none	1.38
(113)	PEI	N/A	N/A	cross-linked	macroscopic solid fibers	0.37

### 2.3 Organic Porous Polymers for DAC

**Figure 8:**
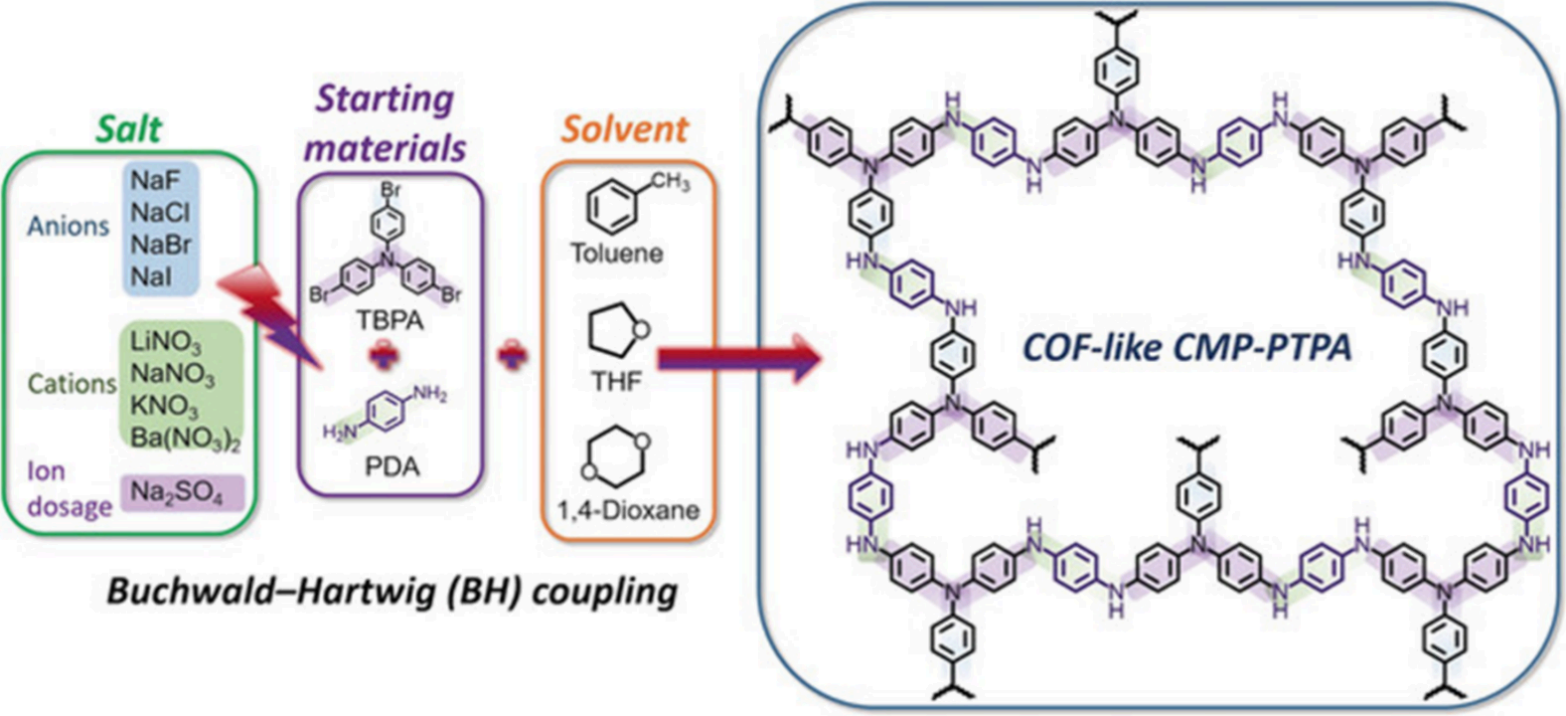
Illustration of poly(triphenylamine) networks through Buchwald-Hartwig coupling. Reproduced with permission from Faul et al.^125^ Copyright 2019 Wiley Publishing.

An important and emerging subsection of amine-containing polymers for DAC are porous organic polymers (POPs). POPs are porous polymeric networks with high surface areas and relatively uniform pore sizes, which are formed through connecting different covalent bonds between organic components.^114^ Many POPs can be amorphous in nature, but crystalline POPs are also available, including covalent organic frameworks (COFs), and can be considered as organic analogues to MOFs. The tunable structures, excellent stability, and ease of synthesis have enabled the use of POPs in various applications such as catalysis,^115,116^ sensing,^117,118^ and CO_2_ capture.^119−121^ Particularly, high surface areas and tunable pore surface chemistries enable POPs to uptake CO_2_ with high adsorption capacities through a physisorption mechanism, much like CO_2_ capture in MOFs, but they can also be functionalized with various amine functionalities to enhance their adsorption capacities through introducing chemisorption mechanisms; this method can also be carried out with MOFs, but as the focus of this review is on organic polymer systems, they will not be discussed here. Instead, the reader is directed toward multiple excellent works if they are interested in MOF-based sorbents for DAC.^122−124^ POPs can be functionalized with amines through various methods, including the use of monomers that contain amine functionalities, postsynthesis functionalization, and impregnation of the POPs with amines or aminopolymers, similar to other solid supports. As an example, Faul et al. demonstrated the synthesis of microporous poly(triphenylamine) netwroks using tris(4-bromophenyl) amine as a “core” molecule and phenylene diamine as a “linker” molecule (Figure 8).^125^ Specfically, Buchwald-Hartwig coupling, which is a palladium-catalyzed cross-coupling method of reacting aryl amines with aryl halides was used for the formation of C–N bonds and development of porous network. Importantly, the incorporation of salts with varying ionic radii during synthesis provided the ability to control the pore properties of the resulting POPs. The surface area of the materials could be modulated from 58 m^2^/g to 1152 m^2^/g simply by altering the salt identity, which directly impacted the sorption capacity of the sorbents. As anticipated, the sorption capacity generally increases with enhanced surface area and pore volume, reaching a maximum value of 3.60 mmol/g at 1 atm in this study.

Additionally, Li et al. synthesized POPs using 1,3,5-triethynlbenzene as a core and 2-amino-3,5-dibromopyridine as an amine functionalized linker molecule.^126^ The core and linker were polymerized through the Sonogashira-Hagihara coupling with the presence of palladium catalyst. By varying the ratio of core to linker, the properties of the POPs can be drastically altered. The surface areas of the materials could be tuned from 17 m^2^/g to 760 m^2^/g and distinct differences in their macroscopic morphology were observed. Specifically, increasing the ratio of linker resulted in a transition from particle with sphere-like morphologies, to uniform tubular structures, which exhibit the highest surface areas. In turn, these materials achieved the highest sorption capacity of CO_2_. While these systems based on aromatic amines exhibit good adsorption capacities and a potential for high cycle stability, their synthesis can be complex and high cost; for example, the use of expensive catalysts, such as palladium, may limit their application for scaled use. To increase cost efficiency, an alternative system has been demonstrated, involving the synthesis of POPs using 1,3,4-trihydroxybenzene and terephthalaldehyde, as the core and linker chemicals, respectively. The only catalyst required to prepare these phenolic-resin type POPs is aqueous hydrochloric acid, which is low cost and widely available. The resulting POPs prepared from this method can contain a wealth of free hydroxyls which can be leveraged as active sites for post functionalization with amines. Zhou et al. demonstrated the synthesis of amine functionalized POPs using this technique.^127^ They investigated multiple different methods to tether amines to the framework and found that reacting the hydroxyl functionalities with bifunctional molecules containing the same or similar bonding sites, such as 1,2-dibromoethane, resulted in reduced amine substitution as both reactive sites would be consumed by the free hydroxyl groups. Alternatively, reacting POP with epichlorohydrin installed epoxide functionalities which could be functionalized with alkylamines to promote interactions with CO_2_ molecules. This work found that the installation of amines resulted in a significantly increased sorption capacity of the sorbent for DAC, even though a decrease in surface area from 703 to 229 m^2^/g was observed. Additionally, the sorbents exhibit no significant performance degradation over 50 regeneration cycles, further exemplifying the potential of these materials to be applied as cost-effective solutions for DAC.

## 3 DAC by Ionic Functional Groups in Polymers

The use of interactions between ions and CO_2_ has been widely employed in many DAC technologies. For instance, aqueous hydroxides were one of the pioneering material solutions for DAC, now becoming a key component of industrial DAC solutions for companies including Carbon Engineering.^128^ Generally, this relies on the chemical reaction between the atmospheric CO_2_ and an ionic species from the dissociation of the hydroxide, to ultimately form a carbonate precipitate, potentially allowing further CO_2_ storage and utilization.^129^ However, similar to aqueous amine systems, the large heat capacity of the water used to make the aqueous hydroxide solutions renders the regeneration process highly energy intensive, necessitating an alternative method to enable more cost- and energy-efficient DAC technologies. A promising candidate for CO_2_ capture via leveraging ionic functionalities is ionic liquids. Briefly, ionic liquids are organic salts with melting points below 100 °C, which are nonvolatile, noncorrosive, and both chemically and thermally stable.^130^

Early study of CO_2_ solubility in ionic liquids includes work from Blanchard et al. in 1999, who found that CO_2_ is very easily dissolved in ionic liquids at room temperature, while ionic liquids remain insoluble with the presence of high concentration CO_2_ (i.e., chemically stable), which can be important for long-term stability considerations.^131^ Additionally, ionic liquids can have tunable molecular structures to allow excellent CO_2_ selectivity and are generally nonvolatile. The nonvolatility of ionic liquids allows them to be regenerated with lower energy requirements and reduced potential for the release of environmental toxins, which may occur in some aqueous amine systems. For example, work done by Blanchard et al. has now become one of the most conventional ionic liquids for DAC application, using 1-butyl-3-methylimidazolium hexfluorophosphate. This molecule can achieve a CO_2_ adsorption capacity of 0.75 mol CO_2_/mol of ionic liquid at room temperature and under a pressure of 8.3 MPa. This work has then led to the investigations of many other ionic liquids with varied cation and anion identities, of which the anion typically plays a dominant role in determining the CO_2_ sorption capacity. Specifically, Brennecke et al. studied ionic liquids containing imidazolium, ammonium, and pyrrolidinium cations with tetrafluoroborate, hexafluorophosphate, or bis(trifluoromethylsulfonyl)imide anions.^132^ It was found that the CO_2_ sorption performance of the ionic liquids containing bis(trifluoromethylsulfonyl)imide anions were not significantly changed by altering the identity of the cation. In contrast, the bis(trifluoromethylsulfonyl)imide anion and hexafluorophosphate anions can significantly impact the solubility of CO_2_ within the ionic liquids. While the anion dominates the CO_2_ sorption capacity of ionic liquids, incorporating cations, which are fluorinated or contain ester groups, can also result in improved performance for CO_2_ adsorption.^133−135^ On the basis of the sorption mechanism of ionic liquids for DAC, a wealth of new polymeric materials have been developed for enabling further improved performance. These materials include poly(ionic liquid)s (PILs), ion exchange resins, and a variety of other polymers containing ionic functionalities such as quaternized biopolymers.^136−140^ This section discusses ion-containing polymer design criteria for enhancing their CO_2_ sorption capacity, optimizing regeneration condition and stability, as well as the potential for reducing production costs in these ion-containing systems.

### 3.1 Tuning CO_2_/Polymer Interactions via Ionic Group Identity

In the case of ion containing polymers, CO_2_ sorption most commonly relies on the use of polymers containing cations of ammonium and imidazolium ions,^141−145^ with a few examples exploring other cations such as pyridinium and phosphonium.^146,147^ There is a wealth of literature examples employing each of these cation identities in varied polymer structures including PILs,^148−150^ ion exchange resins,^151,152^ and other cross-linked porous polymers.^153,154^ Importantly, the identity of these cations directly impacts the CO_2_ capture capacity of the resulting polymeric sorbents.

**Figure 9:**
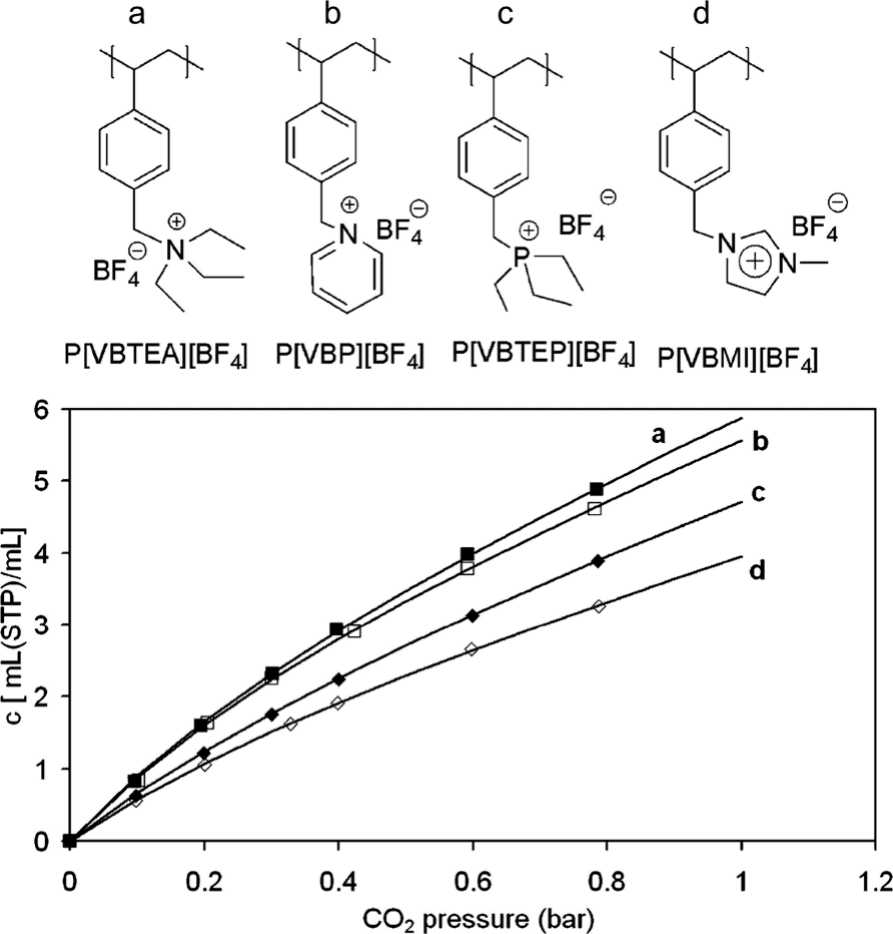
Chemical structures of PILs synthesized with polystyrene backbones containing (a) ammonium, (b) pyridinium, (c) phosphonium, and (d) imidazolium cations along with their corresponding CO_2_ adsorption performances at room temperature up to 1 bar of CO_2_. Reproduced with permission from Sun et al.^155^ Copyright 2009 American Chemical Society

To illustrate the impact of cation identity on CO_2_ adsorption, Sun et al. synthesized a library of PILs containing polystyrene backbones in which the aromatic ring of the polystyrene repeat unit was functionalized with cations and counterions of varied chemical identities.^155^ Their structures, as well as the CO_2_ capture performance, are provided in Figure 9. The majority of these polymers were synthesized using *p*-vinyl benzyl chloride as a monomer which was first functionalized with the desired cation and anion prior to free radical polymerization. The difference in the CO_2_ adsorption isotherms of the polymers is significant even at low CO_2_ pressures where the ammonium and pyridinium PILs have similar performance, and the phosphonium and imidazolium PILs exhibit decreased CO_2_ adsorption capacities. At high CO_2_ pressures, these differences become further pronounced. It is important to note that while these experiments are not under DAC conditions, they exemplify the effect of the cation identity, which can be extrapolated to DAC conditions. More specifically, this work determined that, with the same polymer backbone and counterion type, the affinity of the cations for CO_2_ sorption was highest in ammonium-based cations and decreased in the following order: ammonium > pyridinium > phosphonium > imidazolium. Similar phenomena were observed in other research works using other polymer backbones such as poly(methyl methacrylate).^156^ It was suggested that there are two major factors, including positive charge density in the cation and the hybridization of the cation which can facilitate adsorption of CO_2_ to occur. For example, the ammonium ions have a highly localized positive charge density which can enhance interactions between the polymer and CO_2_ molecules, while other ions (such as imidazolium) can delocalize the positive charge and thus weaken the ability to uptake CO_2_. Similarly, the sp^3^ hybridization of ammonium cations enables them to rearrange and facilitate their access to uptake CO_2_ molecules, while the sp^2^ hybridization in aromatic cations like pyridinium and imidazolium cations can hinder their ability to accommodate CO_2_ molecules. It is also worth noting that while this portion of the discussion has primarily focused on PILs, the same design criteria apply to other DAC systems including some ionic biopolymers as well as other polymers in the literature.

In addition to the cation identity of the ionic polymers, the associated anionic counterion can also impact materials performance for CO_2_ adsorption, including their regeneration ability as well as the CO_2_ sorption capacity. The effects of a few common anions have been previously demonstrated through an atomistic simulation study on a series of imidazolium-based PILs with varying anionic counterions.^157^ Specifically, bis(trifluoromethylsulfonyl)imide ([TF_2_N]^−^), thiocyanate ([SCN]^−^), hexafluorophosphate ([PF_6_]^−^), and chloride ([Cl^–^]) ions in 1-vinyl-3-duylimidazolium ([VBIM]^+^) derived PILs were all investigated. Monte Carlo and molecular dynamics methods were used to simulate the effect of the varied anions on both the diffusion of CO_2_ throughout the PIL as well as its sorption, in conjunction with the structure, dynamics, fractional free volume, and void size distributions of the PILs. Through studying the radial distribution functions of CO_2_ molecules with respect to various atom types within the PIL sorbent, it was determined that the CO_2_ molecule had the strongest preferential interaction with the cationic component, while altering the identity of the counterion only results in limited changes in their ability to associate with CO_2_ molecules. However, this is only the case for counterions that do not actively react with CO_2_. It has been demonstrated that incorporating various organic counterions such as hydroxides, carbonates, acetates, can greatly increase the ability of the sorbent to uptake CO_2_ molecules, depending on the basicity of the anion and its potential to react with CO_2_. For instance, Lackner et al. demonstrated that exchanging the counterions of a commercial anion exchange resin containing ammonium cations and chloride anions with hydroxide or carbonate anions greatly alters the characteristics of the material for DAC.^137^ The main difference is the thermodynamic sensitivity to water during adsorption process, which can be leveraged for developing their respective regeneration methods, an important concept which will be discussed in the next section, while the ability of the sorbents to adsorb CO_2_ also increases. Similar phenomenon has also been reported in many DAC systems in the literature, including quaternized chitosan/PVA aerogels,^136^ hyperbranched polymers,^158,159^ and porous polymers synthesized through emulsion polymerization.^160^ The enhanced adsorption is a result of the ability of the hydroxide ([OH]^−^) and carbonate ([CO_3_]^2–^) anions to react with CO_2_ molecules through the following reaction mechanisms:
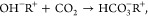
1
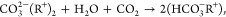
2
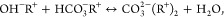
3where eq 1 depicts the reaction of the hydroxide ion bound to the ammonium cation with a CO_2_ molecule to form a bicarbonate. Similarly, eq 2 details the reaction of two carbonate counterions reacting with a CO_2_ molecule to form two bicarbonates. As shown in the reaction schemes, the presence of water is required for this reaction to occur. Additionally, eq 3 represents the equilibrium between the hydroxide, carbonate, and bicarbonate species in the presence of water. At low water concentrations, the hydroxide ions can continue to react until they are completely consumed.

It is worth noting that varying the identity of the ions within the polymer can not only greatly impact the affinity of the system for CO_2_ sorption, but also alter the physical characteristics of the ionic polymers.^161^ Simulation studies have determined that the identity of the anion could affect free volume in the sorbent, which could result in multiple changes of physical properties of the polymer sorbent matrix.^157^ Experimentally, large fluctuations in glass transition temperature (*T*_g_) and viscosities in ionic polymers have been linked to different counterion types, which could alter the ability of CO_2_ to diffuse through the system and the accessibility of the active cationic sites for sorption.^39^ Altogether, designing an ionic polymer containing sp^3^ hybridized, flexible active sites with localized positive energy density, and typically ammonium cations, could be desired for enhancing their interactions with CO_2_ molecules; introducing counterions of hydroxides or carbonates into polymers can improve their CO_2_ sorption performance significantly. Additionally, physical characteristics of the ionic polymers, such as their viscosity, *T*_g_, and free volume fraction are important factors to control how CO_2_ molecules diffuse and react with DAC polymer matrix.

It is important to acknowledge that the macroscopic morphologies of the ionic polymers can influence their ability to adsorb CO_2_. This has been illustrated by He et al. who prepared ionic polymers using a number of different support/templating materials and investigated their respective CO_2_ capture performances.^158−160^ These materials included quaternary ammonium hydroxide functionalized commercially available membranes, carbon black (surface area of 21 m^2^ g^–1^), porous polymeric materials based on colloidal crystal templates (surface area of 17 m^2^ g^–1^), and high internal phase emulsions (HIPE, surface area of 6.2 m^2^ g^–1^, average pore size of 0.83 μm). The colloidal crystal templated and the HIPE based materials exhibited significantly improved adsorption kinetics compared to commercially available materials containing similar functional groups. Among them, the HIPE templated material (adsorption rate: 1.1 × 10^–1^ mmol g^–1^ min^–1^; desorption rate: 3.3 × 10^–2^ mmol g^–1^ min^–1^) outperformed the others with a 12 times greater overall rate of CO_2_ capture/release. The improved sorption rates were attributed to the large, well-connected pores in the porous scaffold prepared by templating methods, facilitating rapid transport of water and CO_2_ molecules. These findings further highlight the significance of accessibility of chemical groups and connectivity of pores within the scaffold for controlling DAC kinetics. Another study by Shi et al. elucidated the influence of pore size, cation spacing, and surface hydrophobicity of the sorbents on CO_2_ capture efficiency.^162^ Through quantum mechanics simulations to calculate ion hydration energy, this work suggested that the use of supporting materials with small pore sizes, short cation distances, and hydrophobic properties could lead to high CO_2_ capacities. It is important to note that there is also potential for enhanced adsorption capacities through the incorporation of both ionic and amine functionalities within the same sorbent. Although this concept is relatively underexplored in the literature, Ke et al. developed polyamine-functionalized imidazolyl PILs for use as heterogeneous catalysts for converting CO_2_ into cyclic carbonates.^163^ While the CO_2_ adsorption capacities of these materials were not explicitly investigated, the PIL containing amine functionalities exhibited faster reaction rates and greater conversions which could suggest enhanced interaction with CO_2_. This study indicates the potential for leveraging these synergistic effects of different functional groups, while designing integrated systems of CO_2_ capture and conversion in the future.

### 3.2 Optimizing Sorbent Regeneration Conditions through Molecular Design

The ability to release/desorb CO_2_ for the sustained use of DAC sorbents is critical to increase their commercial feasibility by reducing material and operational costs. As discussed in the previous section, the most common method for sorbent regeneration is through the application of heat. At elevated temperatures, the interactions between CO_2_ molecules and ionic polymers are significantly weakened, which allows the desorption of guest molecules and regeneration of sorbents. Additionally, CO_2_ can be desorbed from ionic polymers through the application of mechanical forces, such as using a pressure swing.^164^ However, as one can anticipate, applying heat or mechanical force can require high cost and energy consumption, thus making the DAC process less economically feasible. In pervious works from Lackner et al., they demonstrated a desorption method for ion-containing CO_2_ sorbents simply through the introduction of water.^137,165,166^ Specifically, their first work focused on a commercial anion exchange resin containing quaternary ammonium cations for CO_2_ adsorption along with chloride counterions.^137^ The chloride counterions were exchanged prior to adsorption experiments with either hydroxide or carbonate counterions by soaking the resins in sodium hydroxide (NaOH) or sodium carbonate (NaCO_3_) solutions. Exchanging the chloride ions for hydroxide and carbonate ions enabled increased adsorption of CO_2_ from atmospheric concentrations and also induced a thermodynamic sensitivity to water, which could be used as a desorption mechanism. Upon completion of the adsorption reactions (as illustrated in eqs 1–3), the equilibrium between bicarbonates and carbonates can be favored toward the formation of carbonate ions through the addition of water and subsequent desorption of CO_2_, which is depicted by eq 4.
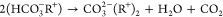
4

**Figure 10:**
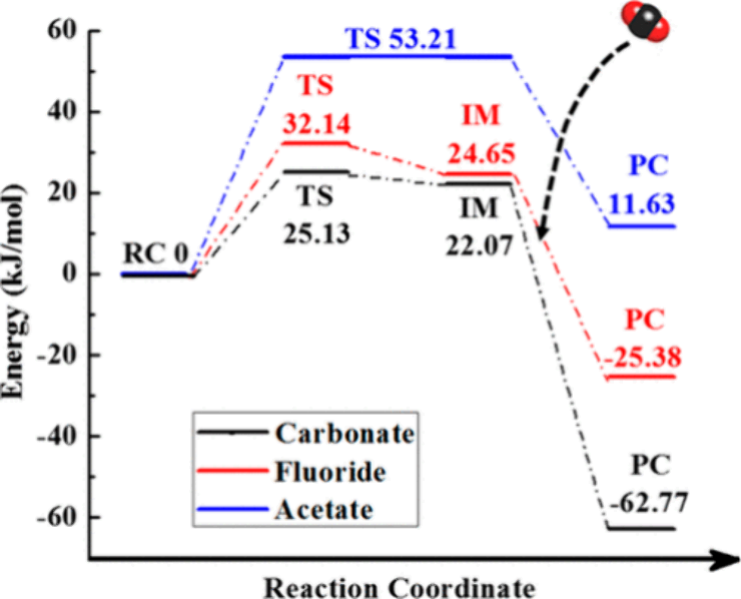
Reaction pathways and the corresponding potential energy profiles for CO_2_ adsorption by PILs with different anions. RC, TS, IM, and PC are abbreviations for reactant complex, transition state, intermediate state, and product complex, respectively. The PILs with carbonate or fluoride adsorb CO_2_ via two steps with an intermediate state, while the PIL with acetate adsorbs CO_2_ via one step with a high activation energy. Reproduced with permission from Luo et al.^167^ Copyright 2017 American Chemical Society.

This process provides a fundamental mechanism behind moisture swing adsorption-desorption and has great potential as an energy efficient and low-cost method for regenerating sorbents in DAC applications. The effect of the anion identity on the moisture swing capability of PIL-derived sorbents containing carbonate, fluoride, and acetate was further investigated using density functional theory (DFT) calculations; the example reaction pathways are provided in Figure 10.^167^ PILs with fluoride and carbonate anions can adsorb CO_2_ through a two-step mechanism, involving two quaternary ammonium cations to react with one CO_2_ molecule. The repulsion between these two cations, which interacted with the carbonate anion or two fluoride anions, could promote the dissociation of hydrated water, making them a better candidate as sorbent materials for capturing CO_2_ from ultralow concentration mixture/source.^168,169^ Moreover, in the case of acetate, CO_2_ was absorbed in one step, suggesting its potential suitability for capturing CO_2_ at a high partial pressure (flue gas capture) because of its large sorption capacity (one acetate anion per CO_2_ molecule) and feasibility for regeneration through conventional approaches (availability of high activation energy). The DFT calculations also determined that the carbonate counterion has the lowest activation energy, greatest affinity for CO_2_, and 2 orders of magnitude greater efficiency for moisture swing desorption of CO_2_ from the sorbent when compared to the systems with acetate and fluoride anions. Building on these findings, Song et al. developed a new humidity-swing absorbent system based on PO_4_^3–^/HPO_4_^2–^/H_2_PO_4_^–^ ions, which can exhibit an 80% higher CO_2_ absorption capacity and much faster kinetics than that of CO_3_^2–^/HCO_3_^–^-based absorbent during moisture swing.^170^ Similar to the conventional CO_3_^2–^/HCO_3_^–^ transformation, PO_4_^3–^ in the ion-exchange resin can be hydrolyzed into H_2_PO_4_^–^ and OH^–^. The hydrolysis reactions generated OH^–^ ions for CO_2_ sorption, and the adsorbed CO_2_ can be released upon increasing the humidity of surrounding environment. The superior performance of PO_4_^3–^ based ion-exchange resin was attributed to the lower free energies (∼20 kcal/mmol in the presence of one water molecule) of PO_4_^3–^ hydrolysis compared to HCO_3_^–^ counterpart. This work presents an alternative strategy for enhancing the CO_2_ capture performance of moisture-swing absorbents. Similar hydrolysis behavior was also found in other anion types, such as S_2_^–^, SO_3_^2–^, and SO_4_^2–^,^171^ suggesting their potential for exploration in moisture swing DAC application.^172^ It was also found that the sorption kinetics of polymers are governed by physical properties such as particle size and microporous structures.^173^ Here we note that despite extensive research on a diverse range of sorbent materials with varying physical structures and chemical compositions, simulations remain to be the primary tool for mechanistic studies; additional complementary experimental investigations can further the understanding of DAC sorbent design principles abilities.^174−176^

**Figure 11:**
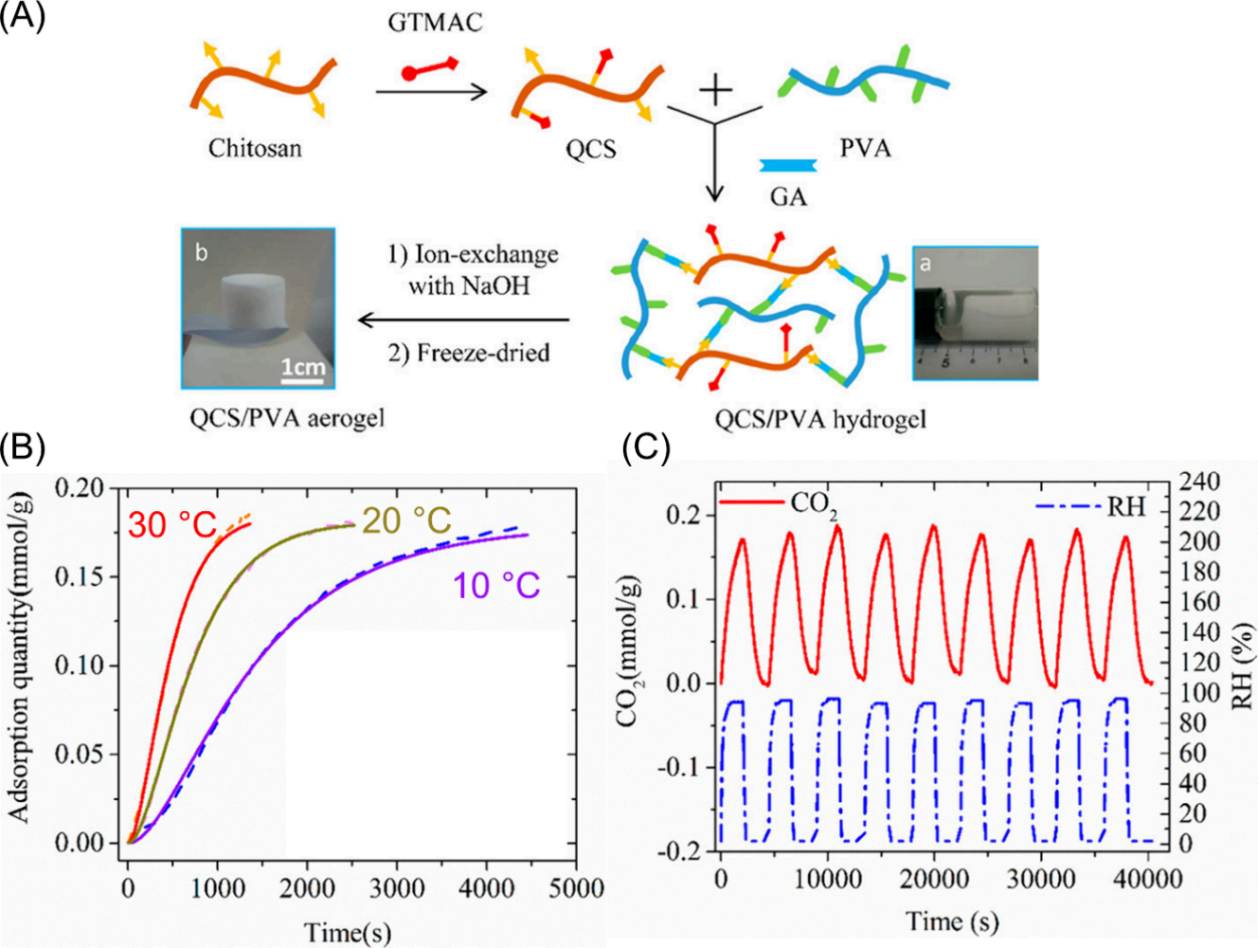
(A) Schematic illustration of the fabrication process for the quaternized chitosan, poly(vinyl alcohol) composite aerogels. (B) CO_2_ adsorption capacities at atmospheric CO_2_ concentrations. (C) Cycle stability of the sorbent at 20 °C over 9 cycles of moisture driven regeneration. Reproduced with permission from Chen et al.^136^ Copyright 2017 American Chemical Society.

Additionally, Chen et al. investigated the ability of a similar moisture swing sorbent system to undergo a series of CO_2_ adsorption and desorption cycles.^136^ Specifically, chitosan functionalized with quaternary ammonium cations and hydroxide anions were investigated for DAC application and their associated adsorption capacity and cycle stability results are presented in Figure 11. The synthesis route of the moisture-swing sorbent is outlined in Figure 11(A). First, chitosan was functionalized through a reaction with glycidyltrimethylammonium chloride (GTMAC) which reacted with amine functionalities in the chitosan backbone, resulting in installation of ammonium cations. The quaternized chitosan was then blended with poly(vinyl alcohol) (PVA) and cross-linked using a glutaraldehyde cross-linker to form a composite hydrogel. The hydrogel then underwent ion exchange with NaOH to replace the Cl^–^ ions with OH^–^ ions and was subsequently freeze-dried to form a porous aerogel.

**Figure 12:**
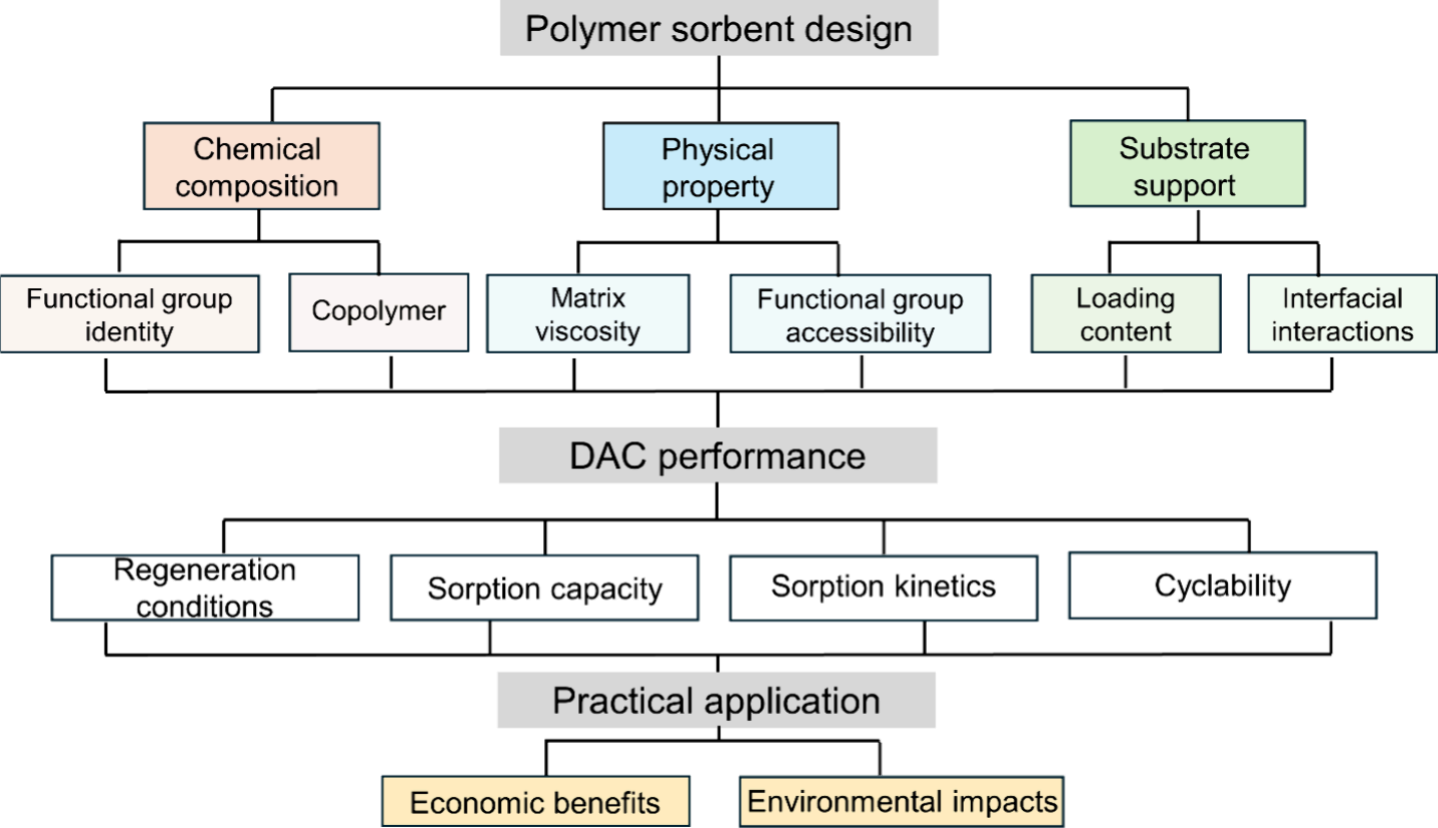
Summary of important aspects of polymer design and their effects on performance in DAC systems.

Figure 11(B) depicts the ability of the quaternized chitosan/poly(vinyl alcohol) composite aerogels to uptake CO_2_ at different temperatures, which indicates that both the equilibrium adsorption capacity and rate can be improved by increasing environment temperature from 10 to 30 °C. At atmospheric concentrations of CO_2_ (∼420 ppm), the maximum equilibrium adsorption capacity is ∼0.175 mmol/g, which outperforms several other ionic polymers for DAC.^158,160^ Importantly, the composite sorbents in this work also exhibit very stable performance over 9 cycles of the moisture swing regeneration. When water is introduced into the system, the adsorbed CO_2_ is exchanged with water molecules, allowing efficient desorption. After the relative humidity is decreased, the aerogel begins to dry, which drives the adsorption of CO_2_. Both the adsorption and desorption behaviors are similar throughout the 9 cycles, indicating that no irreversible side products are formed during the cycling experiments with unaltered physical characteristics of the sorbents (Figure 12(C)). However, it is worth noting that this work employs a quaternized chistosan/PVA composite that is also cross-linked with glutaraldehyde. This is due to the instability of quaternized chitosan itself when exposed to water, providing an excellent example of important material design approach that must be considered aside from the ability of the sorbent to easily undergo desorption. It is also important to note that the potential of these sorbents to be cycled at industrially relevant rates and capacity has still been underexplored. For both cases of polymer undergoing thermally induced desorption of CO_2_ or moisture swing process, performing at lease thousands of cycles is often required for practical applications to reduce operation costs, which could lead to sorbent degradation through thermal degradation, oxidation, leaching, or other potential side reactions. Many research works so far only investigated the stability of DAC sorbent performance over 10–100 s of cycles. To increase the material durability for their long-term use, several methods have been reported, such as through incorporating thermally stable polymers into the design of the DAC sorbents, and/or using cross-linking to enhance both thermal and chemical stability. The regeneration of DAC sorbents which rely on ionic functionalities to adsorb CO_2_ can also be optimized through the incorporation of anions that enables moisture-swing adsorption while also enhancing CO_2_ adsorption capacity. In turn, energy consumption and operation costs can be reduced compared to thermal regeneration, with additional potential for enhanced cycle stabilities.

## 4 Outlook

Design of polymer sorbents for DAC application is an emerging research area for the development of a sustainable society, which has attracted significant research interests and efforts over the past years. Looking forward, there are many exciting and untapped opportunities for studying the fundamental phenomena behind the sorption process of CO_2_ in polymer matrix in DAC technologies, as well as the development of new materials and systems for practical and large-scale applications.

As alluded to throughout this work, polymer physics plays an important role in controlling the performance of DAC sorbents, but changes in polymer properties during the sorption process as well as their associated underlying phenomena are generally underexplored to date. For example, there has been a significant amount of work on understanding SBA-15 supported PEI systems, including polymer chain dynamics and how they alter the sorption performance of the materials.^95,96,103^ These concepts can also be extended to other nanostructured systems such as copolymer-based sorbents; the chemical dissimilarity between distinct segments can drive nanoscale phase separation to occur. For example, block copolymer membranes have been employed for CO_2_ separation. In these systems, soft matrix can be employed to control the gas permeation properties, while the hard domains can provide mechanical integrity for practical applications.^177−179^ Inspired by these results, we envision that the use of block copolymer could also provide a feasible route for DAC, which controlling the chemical identity and composition of different domains may be leveraged to alter the transport and mechanical properties, as well as cycling stability of the sorbents.

Moreover, it has been hypothesized that PEI-derived sorbents are challenged to achieve the theoretical maximum of amine efficiency due to increased viscosity of the polymer as adsorption occurs, suggesting the importance of understanding the kinetics of guest molecule diffusion as a function of CO_2_ concentration in the polymer media, which has not been fully elucidated yet. As the dynamics of PEI within the pores can change upon CO_2_ adsorption and desorption, this offers an excellent opportunity to study the fundamental physics behind these chemically reactive systems. Furthermore, there are many similar areas of study to explore in ion-containing polymers and other amine containing systems. One potential method, among many others, to explore these phenomena is through computational modeling and simulations. Currently, computational studies have been thoroughly leveraged to explore many factors at a system-design level including process optimization, fluid dynamics, and screening of sorbents for improving adsorption capacities.^180−183^ Moreover, there has been significant work in investigating molecular phenomena through both density functional theory studies and molecular level simulations to provide fundamental insight into interactions between sorbents and CO_2_ molecules.^184−187^ Moving forward, similar approaches can be taken to comprehensively investigate the governing physics in these reactive systems.

Additionally, the cost and process scalability are still major challenges for the implementation and practical use of polymer DAC sorbents in industrial relevant scale. The development of simple, cost-effective, and scalable material systems is required for polymers to become economically viable solutions for DAC processes. While PEI is readily synthesized and relatively cheap, the scale at which these polymers need to be produced still outmatches current production volumes of these systems. Similarly, many ion containing polymers, such as PILs, are still generally only produced at laboratory scales. One approach to address this may be through using biopolymers as cost-effective precursors through steps of functionalization to promote their CO_2_ sorption performance. For instance, Hou et al. designed a sorbent derived from cellulose (sourced from bamboo),^140^ which the hydroxyl functionalities on the cellulose repeat units were functionalized with quaternized ammonium ions to develop moisture swing sorbents. While the sorption capacities of these materials were reduced compared to systems like PILs, the authors suggested that their production cost could be much lower.

Another interesting area for further investigations is development of efficient methods for sorbent regeneration, which is important to increase the large-scale feasibility of polymers for DAC; renewable energy resources and natural conditions can be leveraged. As of now, the sorbent regeneration process is one of most energy intensive portions of the DAC process, generally ranging from 0.5 to 18.75 GJ/ton CO_2_ for solid sorbents and 0.62–17.28 GJ/ton CO_2_ for liquid sorbents.^188^ Therefore, methods for reducing energy consumption are highly desired. Using solar, geothermal, and/or other alternative energy sources can offset the CO_2_ emissions associated with regeneration, thus assisting in moving the system design further toward a more carbon-neutral process. Additionally, methods that deviate from conventional temperature or pressure swing desorption can potentially make the regeneration process more energy efficient. Previous works have studied the use of microwave radiation,^189,190^ ultrasound,^191^ magnetic particle,^192^ photoswitching,^193^ and electric swings to assist in reducing energy penalties for regenerating DAC sorbents and suggest excellent opportunities for future technologies.^194^ Similarly, the integration of DAC technologies with processes to convert CO_2_ into useful products is important to increasing the economic viability of these systems, potentially eliminating large needs of carbon storage and transport. We note that CO_2_ can be employed as a building block to prepare products including methane, ethanol, methanol, and carbonates with high atom economy.^120,195,196^ The versatility of these processes enables immense opportunity for increasing economic viability of DAC systems if they can be incorporated into existing technologies.

Geographic locations can play a key role in determining the efficiency of desorption systems. Air pressure, CO_2_ concentrations, humidity, and temperature all vary greatly across the globe, and fully elucidating the effects of these conditions is required for optimizing DAC systems and allowing their informed design and use. If CO_2_ conversion processes are also incorporated into DAC technologies, then the ability and capital cost to transport the upconverted products to various locations must also be considered as many DAC plants are currently in remote locations. Additionally, a common concern with DAC plants is the effect of locally reduced CO_2_ concentrations on vegetation and various other biological processes, which also potentially needs further study. Reduced CO_2_ concentrations near DAC plants would also reduce efficiency of DAC processes, and therefore, the footprint of each plant, as well as the position of plants with respect to each other, should all be fully considered.

Finally, a brief summary of these opportunities is provided in Figure 12. As discussed throughout this work, the performance of polymer-derived sorbents in DAC applications is a complex interplay between the chemical composition and physical properties of the polymers, as well as their interaction with solid supports. Within these design spaces, functional group identities can be optimized, potentially through the development of copolymers with synergistic effects between amine and ionic functionalities, or potentially ordered nanostructures. Additionally, understanding the governing mechanisms behind the physical properties of the polymers can provide insight into optimizing matrix viscosity and functional group accessibility, in addition to interfacial interactions with the solid support to enhance CO_2_ adsorption. Improving all of these factors can elevate the efficiencies DAC technologies through increased sorption capacities, kinetics, and cyclability, while also reducing energy required for regeneration. Altogether, advancement in the field of DAC technologies through these avenues can enable minimize negative anthropomorphic environmental impacts while maximizing economic benefits.

## 5 Conclusions

Increased concentrations of CO_2_ in the atmosphere caused by human-related activities are linked to various negative consequences, including more severe natural disasters, and rising ocean levels. As a society, it has become imperative to move toward a carbon neutral society to prevent further increase in atmospheric CO_2_ concentration which requires transitioning to alternative fuel sources that do not require the emission of CO_2_, as well as developing technologies and processes to directly remove CO_2_ from our atmosphere. Polymers have played a key role in developing DAC sorbents for DAC and possess many opportunities to further optimize their performance. This work summarizes amine-containing and ion-containing polymers, which are the most studied polymer systems for direct air capture (DAC) of CO_2_. Specifically, many properties of amine containing polymers including amine functionality, polymer molecular weight, and topology, can impact their performance as DAC sorbents. The effects of these characteristics, as well as interactions with solid supports, are discussed to provide a perspective on potential avenues for further technological improvement. Similarly, ion identities in ion containing polymers directly dictate their ability to reversibly uptake and release CO_2_; these effects are fully described. Overall, this work provides a comprehensive review on polymer material design principles for DAC, as well as future research opportunities in this emerging and important area for the development of a sustainable environment and society.
